# A case study of a national ethics committee: challenges in regulating HIV prevention research in Zimbabwe

**DOI:** 10.1186/1742-4690-9-S2-P232

**Published:** 2012-09-13

**Authors:** ME Phiri-Shana, R Musesengwa, S Ruzario, RB Gutsire-Zinyama, R Gunda

**Affiliations:** 1 Medical Research Council of Zimbabwe, Harare, Zimbabwe

## Background

As efforts in search of effective HIV prevention strategies intensify, studies have become exceedingly complex, posing several regulatory challenges to most African Ethics Committees, the MRCZ included. Challenges faced include: Researchers not being clear on how to operate in compliance to local and sponsor country regulations; i.e. when a host country ethics committee has issued a decision on an issue concerning rights, safety and well being of participants while an overseas ethics committee has not. Prevention studies require frequent assessment, at MRCZ this has been hampered by limited resources. Ascertaining comprehension of these complex studies by participants and ensuring they receive new information promptly has also been a challenge. MRCZ has taken several measures to address regulatory challenges faced.

## Methods

Routine, for- cause and passive inspections of ongoing approved research studies have been done. During these, participant comprehension checklists used at enrollment were audited and interviews with participants held to assess study knowledge levels. MRCZ has also set up a database to record types of inspections, number of Serious Adverse Events and protocol deviations

## Results

From year 2000, MRCZ has actively and passively inspected about 1000 studies of these 5 were for- cause. MRCZ has also trained 340 research staff and 15 members of Council in Research Ethics, GCP and National Research Guidelines to build capacity. Due to this there has been: - Adoption of a harmonized review process; A decrease in for- cause inspections and an improvement in study documentation. All initial and continuing review submissions now include GCP certificates of study staff as well as participant comprehension checklists. 90% of interviewed participants show understanding of research studies they are involved in.

## Conclusion

Regulatory bodies should consolidate efforts in understanding the complex and dynamic HIV research arena and in ensuring that research is carried out in a well regulated arena.

